# Intraoperative discrimination of native meningioma and dura mater by Raman spectroscopy

**DOI:** 10.1038/s41598-021-02977-7

**Published:** 2021-12-08

**Authors:** Finn Jelke, Giulia Mirizzi, Felix Kleine Borgmann, Andreas Husch, Rédouane Slimani, Gilbert Georg Klamminger, Karoline Klein, Laurent Mombaerts, Jean-Jacques Gérardy, Michel Mittelbronn, Frank Hertel

**Affiliations:** 1 Centre Hospitalier de Luxembourg, National Department of Neurosurgery, 1210 Luxembourg City, Luxembourg; 2 Medical Faculty, Saarland University, E66421 Homburg (Saar), Germany; 3 University of Luxembourg, Luxembourg Center for Systems Biomedicine, 4362 Esch-sur-Alzette, Luxembourg; 4 Luxembourg Institute of Health, 1445 Strassen, Luxembourg; 5 Laboratoire National de Sante´, Luxembourg Center of Neuropathology, 3555 Dudelange, Luxembourg

**Keywords:** CNS cancer, Medical research

## Abstract

Meningiomas are among the most frequent tumors of the central nervous system. For a total resection, shown to decrease recurrences, it is paramount to reliably discriminate tumor tissue from normal dura mater intraoperatively. Raman spectroscopy (RS) is a non-destructive, label-free method for vibrational analysis of biochemical molecules. On the microscopic level, RS was already used to differentiate meningioma from dura mater. In this study we test its suitability for intraoperative macroscopic meningioma diagnostics. RS is applied to surgical specimen of intracranial meningiomas. The main purpose is the differentiation of tumor from normal dura mater, in order to potentially accelerate the diagnostic workflow. The collected meningioma and dura mater samples (n = 223 tissue samples from a total of 59 patients) are analyzed under untreated conditions using a new partially robotized RS acquisition system. Spectra (n = 1273) are combined with the according histopathological analysis for each sample. Based on this, a classifier is trained via machine learning. Our trained classifier separates meningioma and dura mater with a sensitivity of 96.06 $\pm$ 0.03% and a specificity of 95.44 $\pm$ 0.02% for internal fivefold cross validation and 100% and 93.97% if validated with an external test set. RS is an efficient method to discriminate meningioma from healthy dura mater in fresh tissue samples without additional processing or histopathological imaging. It is a quick and reliable complementary diagnostic tool to the neuropathological workflow and has potential for guided surgery. RS offers a safe way to examine unfixed surgical specimens in a perioperative setting.

## Introduction

Meningiomas (MGM) constitute some of the most frequent tumors of the central nervous system (CNS) with a lifetime-risk of approximately 1%^1^. They are a diverse group of mesenchymal tumor entities, deriving from arachnoid cap cells^2^.

Radiological diagnostics of meningiomas is usually accomplished either by magnetic resonance imaging (MRI) or computed tomography (CT) scan. The gold-standard of treatment is the complete surgical resection. Complementary approaches can be radiosurgery/radiotherapy, or in some cases application of hormonal treatment^3,4^ or immunotherapy^5^. After successful surgical resection, the recurrence rate is between 7 and 25% for benign (WHO I), 29–52% for atypical (WHO II) and 50–94% for anaplastic meningiomas (WHO III)^1,6^. The recurrence rate negatively correlates with the extent of resection^2,7^. The main goal of surgical resection should always be a Simpson-grade I resection, concomitant with a symptomatic 10-year recurrence of 9%. It is often difficult to exactly distinguish the tumor border or differentiate infiltrated dura mater from adjacent healthy tissue^2^. Therefore, a technical support could be helpful to complete the tumor removal. Intraoperative neuropathological diagnostics (smear or snap frozen) arrive time-delayed, are invasive and rarely used in meningioma surgery. An intraoperative MRI or CT scan is time-consuming and associated with logistic challenges as well as high costs^8^. Several spectroscopic techniques such as infrared and nuclear magnetic resonance spectroscopy^9,10^ as well as Raman spectroscopy (RS) are under investigation for this tissue diagnostics, especially the latter shows a great potential for tumor differentiation^11–22^. The Raman effect occurs during the interaction with the specific vibrational mode of molecular functional groups, when photons either gather or loose energy (Stokes and Anti-Stokes effect). The resulting wavelength shift is characteristic for the vibrational mode of the underlying molecule. Thereby, heterogeneous tissues with a huge diversity of molecules result in characteristic complex spectra as a function of their biochemical compositions^23^. With Raman Microscopy, a differentiation between snap-frozen meningioma tumor tissue and normal dura mater tissue was reported^24^. Even RS of blood samples alone was described as suitable to identify meningioma patients by detection of specific spectra features^25^. Several recent studies have demonstrated the potential of RS in oncologic diagnostics^9,11,24–26^. However, there has been no report on the intraoperative application of RS for the diagnostics of meningiomas by help of macroscopic samples. In contrast to microscopic RS-techniques, non-microscopic RS could be used in situ without any further tissue processing. Handheld probes suited for intraoperative neurosurgical use of RS have been recently developed^14^. Therefore, this technology bears the potential for direct intraoperative resection control.

The Raman spectrometer applied in this paper (Solais, Synaptive, Toronto, Canada) offers the possibility to document in a fully robotized manner the defined measuring points with their exact coordinates on the tissue sample. The system is applicable without any kind of additional histology or tissue processing during the surgical procedure. After integrating it into a handheld probe, it could be used in the surgery cavity itself to indicate tumor borders.

## Materials and methods

Applying the Solais™ Raman spectrometer intraoperatively, we take a close look on meningioma and healthy meningeal tissue and compare their Raman spectra. We use dimension reduction and classifier algorithms such as the t-distributed stochastic neighbor embedding (tSNE) and support vector machine (SVM) classifier^27,28^ for our analysis. Finally, we test the clinical applicability of our trained classifier on an extra sample consisting of dura mater and meningioma. The image thereof is superimposed by a tumor heat map generated by the trained classifier.

### Intraoperative Raman spectrometry

**Figure 1:**
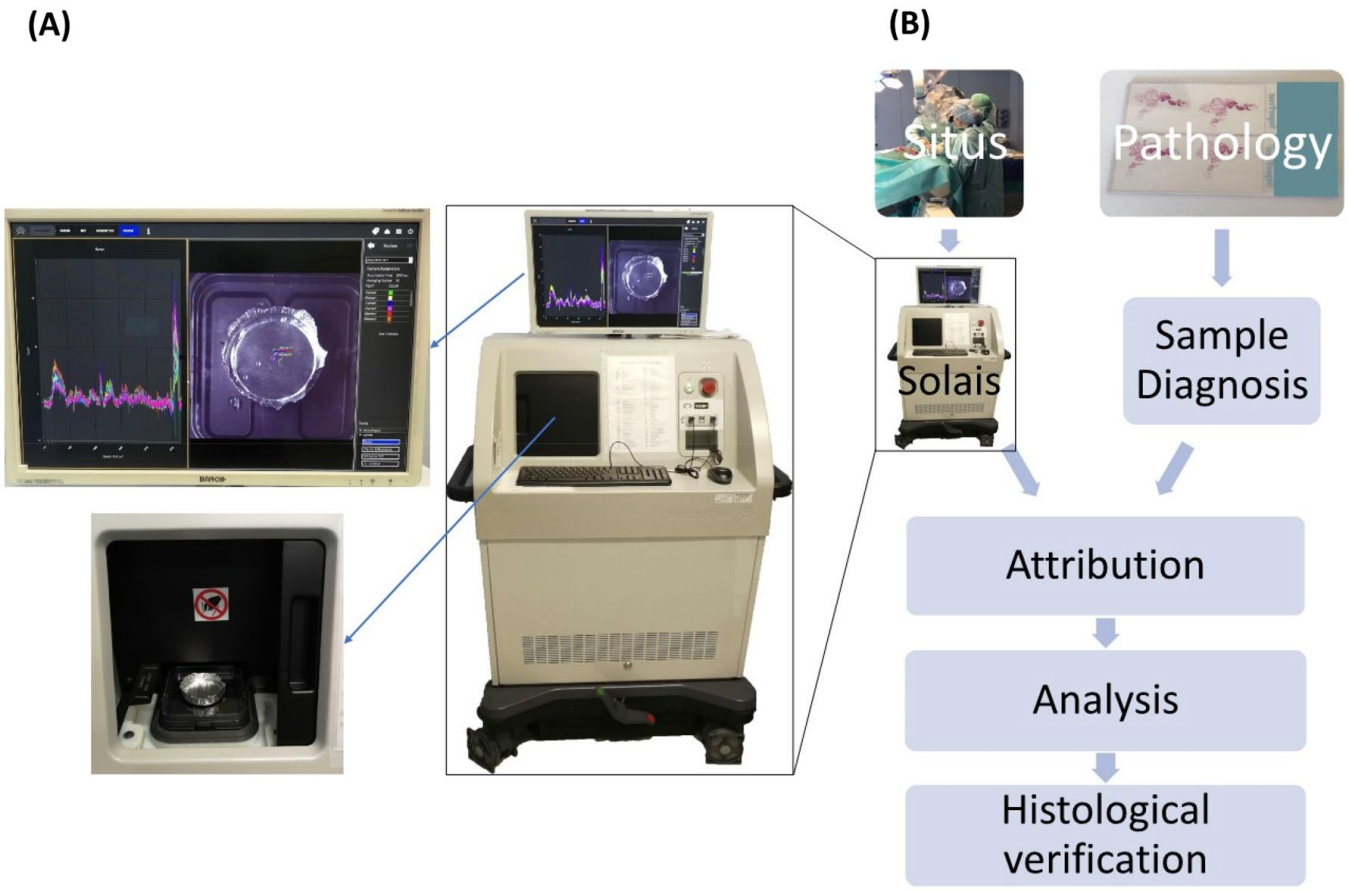
**(A)** Spectroscopic data acquisition and visualization system with robotized measuring spot navigation (Solais, Synaptive, Toronto, Canada). Upper left: Computer screen with the spectral curves on the left side and the white-light image on the right side. Lower left: Shows the robotized measurement chamber. **(B)** Flow chart of data flow.

The device used in our study enables robotized measuring spot navigation, spectral data acquisition and visualization in one unit (Solais, Synaptive, Toronto, Canada) (Fig. 1A). The system comprehends a movable stage, on which the sample can be placed and a visible-light-camera for the measuring point documentation. Furthermore, the measuring device is equipped with a 785 nm laser source operating at 50 mW and an optical coherence tomography (OCT) instrument. A coordination system enables to relocate retrospectively recorded measuring points in the visible light images (VLI) in a precise and reliable way. The data is acquired using an aluminum background due to its negligible inherent Raman spectrum. The average accumulation time was between 800 and 2000 ms with 6 to 30 acquisitions to enhance the signal-to-noise ratio. For each spectrum, the Solais™ records 1603 single values, which are non-linearly mapping to a wavenumber range between 314 and 2994 cm^−1^ with an average resolution of 1.67 cm^−1^.

### Patient data

The INSITU study, Nr. 201804/08, was approved by the ‘Comité National d’Ethique de Recherche’ (CNER), the national ethics board of Luxembourg, and is handled accordingly to the ’EU General Data Protection Regulation (GDPR)’ law from august 1st, 2018 and the study was in accordance with WMA Declaration of Helsinki-Ethical principles for medical research involving human subjects^29^. Each recruited patient gave written informed consent.

Table 1 summarizes the statistics of all 59 patients, 1273 measuring points of 223 samples. Percentages in parentheses correspond to the training or validation set portions within the totality of the samples or the measuring points. We analyzed meningioma tissue from 48 patients as well as non-infiltrated dura mater from 11 patients of the same group. Dura mater samples were further analyzed from 11 patients that were operated for other pathologies (1 astrocytoma, 1 oligodendroglioma, 2 glioblastomas, 1 leiomyosarcoma, 5 metastases, 1 schwannoma). Within the meningioma group, 13 (36%) patients had transitional, 13 (36%) meningothelial, 4 (11%) atypical, 4 (11%) fibrous, 1 (3%) not-otherwise-specified and 1 (3%) secretory meningioma. One appropriate anaplastic meningioma sample with macroscopically visible transition from tumor to dura mater was selected for additional histopathological verification as well as for testing the clinical applicability. Therefore, it was densely sampled with 108 individual RS measuring points.

**Table 1:** Data overview.

Meningioma subtype	Patients	Patient’s (n; %)		Measuring points (n; %)	
		Training set	Validation set	Training set	Validation set
**Pure tumor tissue**					
Transitional meningioma	13	10 (76.9%)	3 (23.1%)	171 (74.7%)	58 (25.3%)
Meningiothelial meningioma	19	15 (78.9%)	4 (21.1%)	197 (78.5%)	54 (21.5%)
Atypical meningioma	4	4 (100%)	0	62 (100%)	0
Fibrous meningioma	6	6 (100%)	0	69 (100%)	0
Meningioma, not otherwise specified	2	2 (100%)	0	20 (100%)	0
Secretory meningioma	1	1 (100%)	0	14 (100%)	0
Subtotal “MGM”		**23 (88.5%)**	**3 (11.5%)**	**336 (85.3%)**	**58 (14.7%)**
Healthy dura mater	22	16 (72.7%)	6 (27.3%)	128 (79.0%)	34 (21.0%0
Tumor infiltration zone	Vide supra	Vide supra		371 (80.7%)	89 (19.3%)
Verification sample	1	1		108	
Total				1268	

Three classes were defined: Unequivocal meningioma tumor tissue, healthy dura mater and tumor-infiltrated dura mater samples. The data was split up in a training and validation set. The percentages indicate the corresponding portions of samples and measuring points, subdivided in the different tissue types. Upper table part: Meningioma samples. Middle table part: Origin of the probed healthy dura mater samples. Lower table part: Meningioma-infiltrated dura mater samples. Closing line: Total numbers of patients, samples and measuring points. Notice that, the infiltration zone samples belong to patients that were already considered in the other classes. The kept out surgical-site-simulating verification sample was of atypical meningioma tissue origin and was used for the subsequent classifier mapping study.

Significant values are in bold.

### Data acquisition, labelling and analysis

Figure 1B illustrates the workflow of the study. The tissue is collected intraoperatively. It is kept humid with physiological saline to avoid any tissue destruction due to drying. Before measuring, the tissue is not modified by any further fixation methods, neither chemically (e.g., formalin-fixed, paraffin embedded) nor physically (like cryopreservation). Samples were analyzed within 20 min after excision. The sample is then put on an aluminum dish, which is placed afterwards on the stage inside the Solais™. The output of the Raman-scan are the spectral curves corresponding to the tracked measuring points on the VLI. The tissue samples are afterwards stored, separately, in a formalin solution and sent for neuropathologic analyses. The detailed pathology report allows us to attribute the exact sample diagnosis to every spectrum with tags, considering the major tumor diagnosis and more detailed tissue properties. To avoid biases, the neuropathologist is blinded to the results of RS. The described procedure of the sample acquisition and measurement allows exact retracement and is relatively quick and non-destructive.

#### Data labelling and unsupervised analysis

For analytical purposes, the collected Raman spectra are examined with a custom-made MATLAB™ software. The Solais-preprocessed data (background removal, Savitzky Golay filtering) are checked for artefacts with the aid of an (in house designed) graphical user interface, called RamanLabeler. Outliers and cosmic ray artifacts were removed and excluded from further analysis. The diagnostic information from the pathological analysis of tissue samples is linked to the spectra by manually assigning appropriate tags to each Raman spectrum. Next step is the spectral analysis of the pathologically defined groups. Unsupervised learning techniques are employed for exploratory analyses, applying tSNE^27^ as a dimension reduction cluster visualization tool.

#### Supervised machine learning classification

Before classifier training, the global data is split up in a training and a test set in an approximate ratio of 5:1, considering a patient-wise stratification and equilibrated acquisition parameters for both sides. Both sets have been carefully separated throughout the process avoiding any potential confounding variables. Importantly, the results are robust to the way of splitting (patient-wise or random split) and no pre-processing is required, indicating a high natural separability between MGM and DM. The tSNE was created with the training data set only. Supervised learning is carried out using a support vector machine (SVM) classifier^28^ to predict the class labels (MGM or Dura) from the spectra. For visualization of the trained classifier performance, the VLI of a tissue sample can be overlaid with a posterior class probability heat map output of the classifier. As a case study to determine the suitability in larger tissue contexts, a large representative infiltration zone sample is analyzed. After Raman acquisition, this sample was formalin-fixed, paraffin-embedded and sliced into 21 consecutive hematoxylin & eosin (H&E) sections. Fiducial markers in the form of incisions guide the exact orientation. Because of the uneven sample-surface, the single sections were stacked and fused to map the entire sample with a surface profile. By superimposing the reconstructed surface H&E image with the VLI of the Raman acquisition, the individual RS-scanned points can be validated on a histological level (Fig. 5). The obtained composition image is overlaid with the classifier heat map of the meningioma probability. This way, the result of the classifier can be directly compared to the histology. Given the size of the specimen that is comparable with a surgical site of interest, this approach demonstrates the suitability for intraoperative use.

### Ethics approval

The INSITU study, CNER Nr. 201804/08, is approved by the ‘Comité National d’Ethique de Recherche’ (CNER) on 2018-07-13 and is handled according to the law, state 1st august 2018 ‘EU General Data Protection Regulation GDPR’31, and to the WMA Declaration of Helsinki-Ethical principles for medical research involving human subjects. Each recruited patient gives a written informed consent.

## Results

### Exploratory data analysis

**Figure 2:**
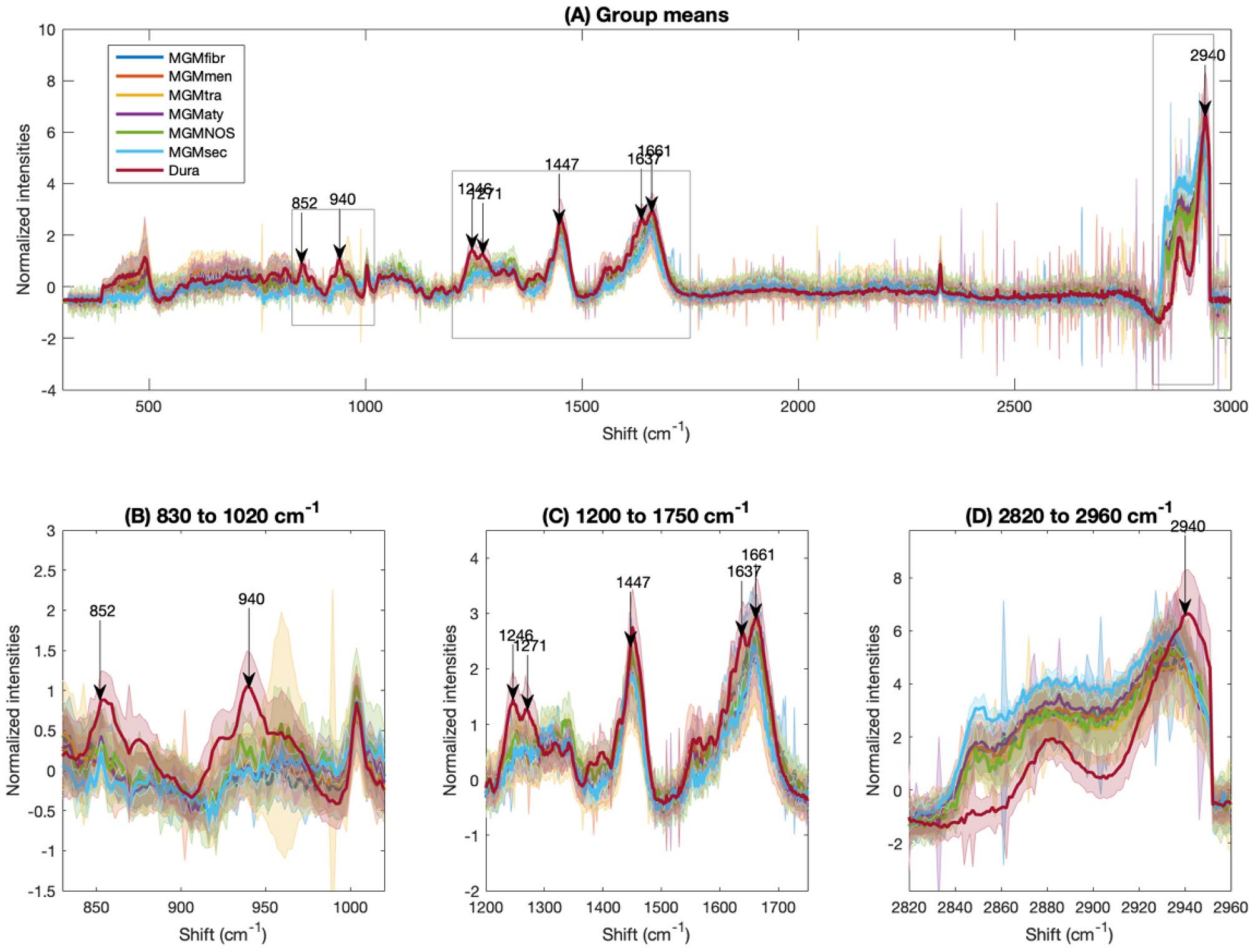
**(A)** Individual average spectra and standard deviation of meningioma subtypes and of dura mater. Based on 47 measuring points of fibrous meningioma (MGM_fibr, dark-blue), 110 of meningothelial meningioma (MGM_men, orange), 159 of transitional meningioma (MGM_tra, yellow), 4 of secretory meningioma (MGM_sec, light blue), 56 of atypical meningioma (MGM_aty, purple), 25 of NOS-meningioma (MGM_NOS, green) and 117 of dura mater (Dura, scarlet). The spectra were z-score normalized. **(B)** to **(D)**: Zoomed-in subintervals with focus on the spectral differences between the different meningioma subtypes and dura mater. **(B)** Subintervals from 830 to 1020 cm^−1^, **(C)** from 1200 to 1750 cm^−1^, **(D)** from 2820 to 2960 cm^−1^. The arrows in **(A)** to **(D)** represent the distinctive peaks between meningioma and dura mater, partially in line with the collagen spectrum^38^.

**Figure 3:**
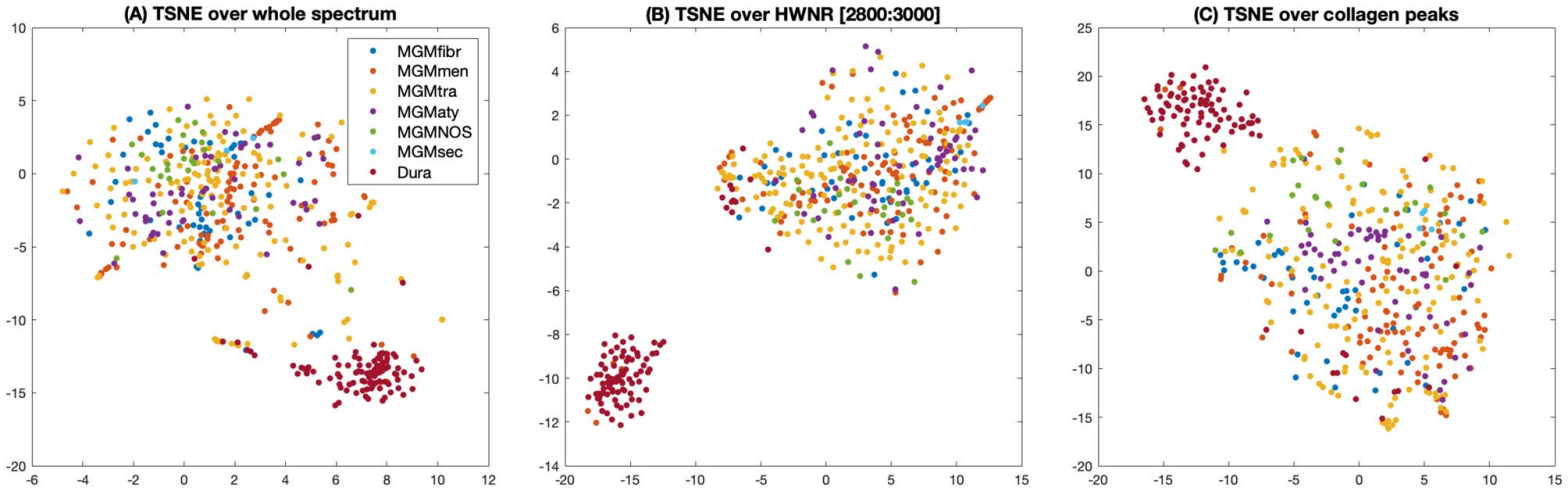
The tSNE-Cluster, based on the training data set, of meningioma subtypes and dura mater. The color-code is the same as in Fig. 2. **(A)**, tSNE-Cluster of pathologically secured diagnoses of entire Raman spectrum of the chosen subclasses. **(B)** tSNE Cluster of pathologically approved diagnoses over high-wavenumber region (HWNR), interval 2800 cm^−1^ to 3000 cm^−1^. **(C)** tSNE Clusters based on collagen peaks.

Figure 2 shows the average Raman spectra of all meningioma subtypes, as well as of dura mater. Differences between normal dura mater and the meningioma subtypes can be observed in the entire spectrum as well as in selected spectral subregions (Fig. 2B–D). The meningioma subtypes share most spectral characteristics when compared to dura mater. It is therefore possible to include all analyzed subtypes into a single class to build the classifier. The surgeon does not need to know the subtype prior to surgery in order to make use of this technique. Reduction to two dimensions using tSNE in Fig. 3 shows two well separable clusters. The first one corresponds to the dura mater measuring points (colored in red), whereas the second one includes all meningioma subtypes (multiple color-coded, except red). This clustering can be reached by the analysis of either the entire spectrum (Fig. 3A) in the high wavenumber region (HWNR) from 2800 to 3000 cm^−1^ (Fig. 3B) or using distinct peaks that are different between the entities (Fig. 3C). We used samples for the classifier training and validation that were homogenous in nature and either only tumor or only dura mater. For verification, we included infiltration zone (Suppl. Figs. S3 and S5) and found that those also clustered with either of the two described clusters in all three spectral sub-analyses.

### Spectral difference analysis

**Figure 4:**
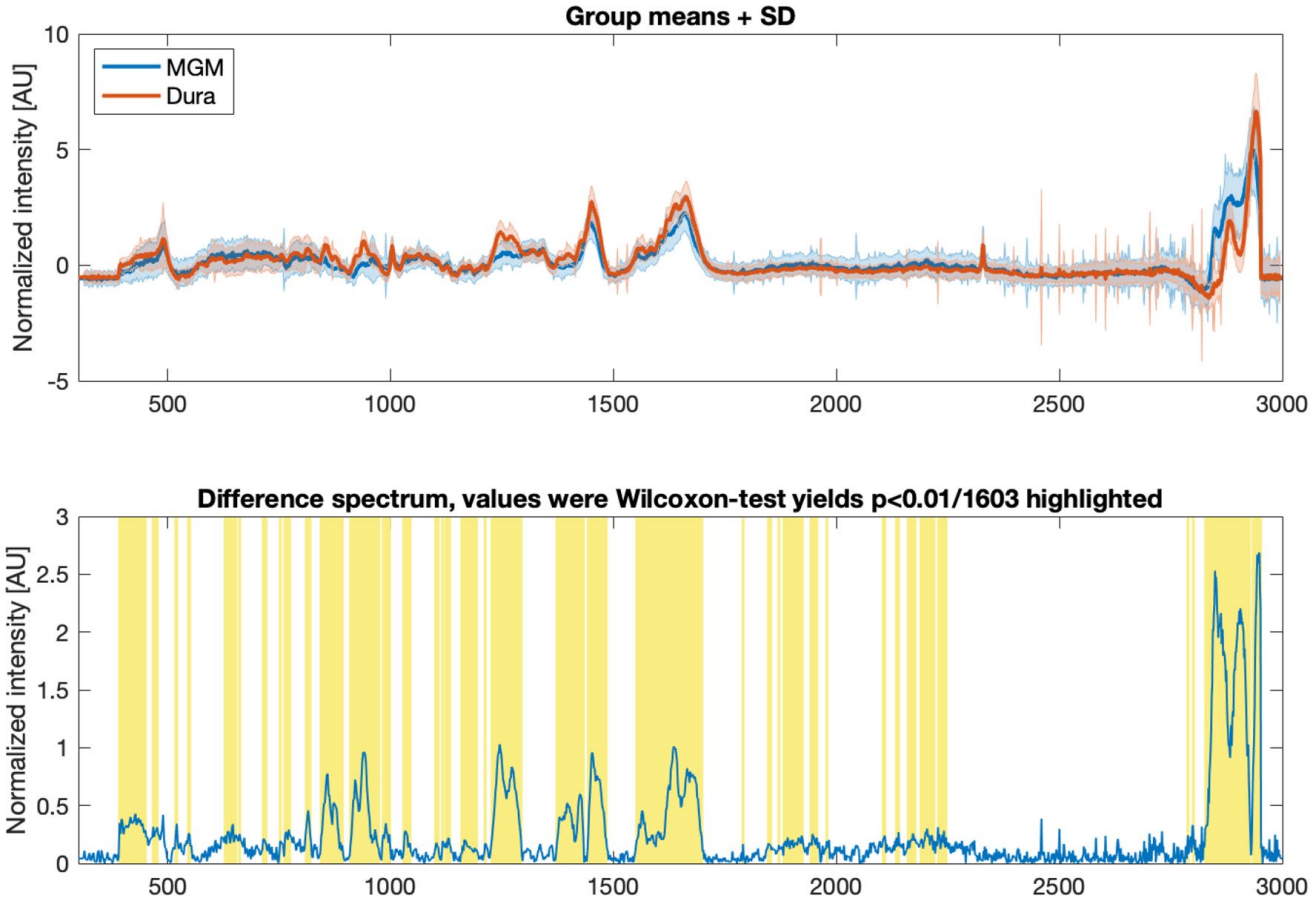
Top: Average spectra of all normalized (z-score normalized) meningioma and dura mater measurements; Bottom: Absolute difference spectrum of the previously plotted average spectra. Significant differences, calculated using Wilcoxon-Mann–Whitney-U test (Bonferroni-corrected p-value, resulting in p ≤ 0.01/1603 = 10 − 6), are highlighted in yellow.

We next wanted to know which areas of the spectrum show significant differences and what may be the underlying tissue composition. Figure 4 quantifies the differences of normalized mean meningioma vs. mean dura spectra. The significantly different Raman shifts, based on a p-value of $p \le 0.01/1603 = {10}^{-6}$ (Bonferroni-corrected Wilcoxon-Mann–Whitney test), are highlighted in yellow. The high wavenumber region (HWNR) between 2800 and 3000 cm ^−1^ shows large and consistent differences between the groups. This area corresponds to the aromatic and aliphatic hydrogens in carbohydrates, indicating differences in the relation of sugar to fatty acids and therefore the relation of cytoplasm to cell membrane, cell size and cell density^20,30^. A tSNE computed on this region leads to a good separation between tumor tissue and dura mater (Fig. 3B).

Several peaks that are characteristic for collagen (815, 855, 876, 938, 1003, 1033, 1250, 1319, 1450 and 1663 cm^−1^)^31^, are of higher intensity in dura mater compared to the meningioma spectra. In fact, if a tSNE clustering is performed solely based on these peaks, meningioma and dura mater separate well (see Fig. 3C). Therefore, the differences in collagen content are an important distinctive feature in the spectral analysis as it is in histopathology.

### Machine learning based tissue classification

In order to train a classifier for tissue discrimination, disjoint classes had to be established. Samples need to be pure in that they do not contain infiltration zones. The first class corresponds to the pure meningioma samples, including all subtypes, and the second represents healthy dura mater. After exclusion of spectra altered by clear cosmic ray artifacts, the test set, the infiltration zone samples and the sample later used for a detailed histological case study, N = 529 spectra (422 meningioma, 107 dura mater spectra) remained for classifier training with a linear support vector machine (Suppl. Table S2). The classification was validated twice, once using fivefold-cross-validation from the full spectra (1603 features per Raman spectrum) and afterwards with the external test set, created by considering a patient-wise stratification.

For the fivefold cross validation, a validation set containing 1/5^th^ of the randomized spectra was split from the data, and the classifier was trained on the remaining 4/5^th^ of the data. The classifier performance was then evaluated on the held-out unseen test set. This procedure was repeated 5 times until all data were used once for testing. The performances of the optimized classifier on the cross-validated dataset are 96.06 $\pm$ 0.03% sensitivity and 95.44 $\pm$ 0.02% specificity (for ROC and PR-curves see Suppl. Fig. S4). The validation with the hold-out external validation data set yielded accuracy values of 100% for sensitivity and 93.97% for specificity.

**Figure 5:**
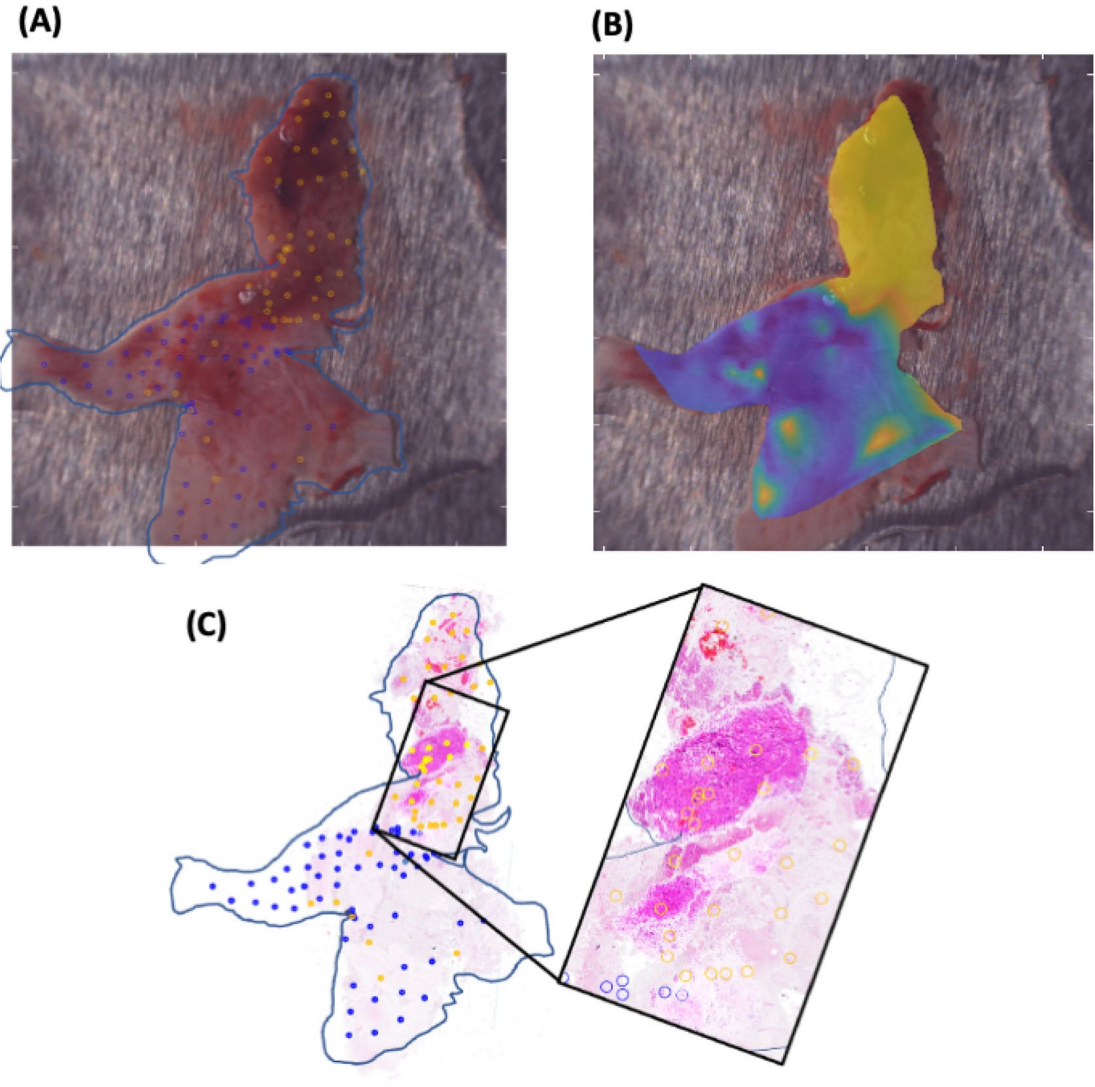
Histological verification of the meningioma-dura mater-classifier. **(A)** Visible Light Image (VLI) exported from the Solais with engraved measuring points and with a retraced frame. **(B)** Same VLI superimposed by a classifier heat map with color scale ranging from blue (not meningioma tissue) to yellow (meningioma). The greenish parts are the transition zones between the meningioma tissue and dura mater. **(C)** Superposition of 21 hematoxylin & eosin stained slides of the same sample with the tumor infiltration zoom-in window and measuring point specifications.

While the training of the classifier relies on small samples containing only one class of tissue, the tool shall be used on larger specimens with mixed content, ideally in situ at a later point. Therefore, we verified the classifier on a histological level with a tissue sample consisting of tumor tissue as well as dura mater, resembling the situation in situ (Fig. 5). Macroscopically, it is impossible to distinguish tumor tissue reliably on this particular sample, however the surgeon needs histological precision of the sample’s nature. The sample was systematically scanned point-by-point and sectioned in the same orientation afterwards. The surface was reconstructed from serial scans and histologically assessed. The classifier identified the tumor, as well as the dura reliably (sensitivity 93.0% and specificity 89.7%). In the infiltration zone, some measurements would be classified either as dura or tumor. Both classes were intermingled accordingly, resulting in some misclassified points where an exact retracement was not possible on the histological section.

## Discussion

In tumor resection, there is a need for a non-destructive intraoperative tool to differentiate between tumor and healthy tissue in surgical disciplines. In meningioma surgery, an important challenge in this context is the differentiation between tumor and healthy non-infiltrated dura mater. Over the last decades, the potential of RS and also of stimulated Raman scattering microscopy for this issue has been increasingly recognized. Koljenovic et al. yield an accuracy of 1.0 for the detection of meningioma infiltration areas within dura mater by Raman microspectroscopy^24^. Krafft et al. apply a similar spectroscopic method comparing it with IR spectroscopy. They identify tumorous features of meningiomas extracted from a Raman map^9^. Focusing on the Raman resonance effect, Zhou et al. are likewise able to discriminate between tumorous and healthy meningeal tissue with a sensitivity of 0.91^26^. Aguiar et al. are able to produce similar results by applying conventional near-infrared RS^11^. All aforementioned studies have in common, that their tissue specimens are cryopreserved and/or histologically processed before the measurements. Orringer et al. developed a prototype for fiber-laser-based stimulated Raman scattering microscopy which combines the advantages of microscopy with RS-based tissue contrast labeling^32^.

Our study shows that RS alone can properly discriminate between meningioma and dura mater with a sensitivity of 96.06 $\pm$ 0.03% and a specificity of 95.44 $\pm$ 0.02% for internal fivefold cross validation and 100% and 93.97% if validated with an external test set. We investigate intraoperative spot measurements on native samples without any kind of tissue processing. In contrast to Raman microscopy, the method can give direct feedback on tumor origin relying on the assessment of the trained classifier which does not require the presence of a neuropathologic expert being familiar with microscopic analyses. This could potentially be a helpful tool for the surgeon for intraoperative resection control.

Furthermore, we analyzed the statistically significant spectral differences between meningioma and dura mater with a special focus on the biochemical composition. Characteristic peaks for collagen show a higher intensity in the dura mater spectra compared to those of meningioma and a simple TSNE plot, based only on the collagen peaks, shows distinct clusters for both entities. We conclude that the collagen content is an important distinctive feature between meningioma and dura mater, also discussed by Protasoni et al.^33^. A closer look into the possible differentiation of meningioma-subtypes based on their collagen content should be examined in further studies.

We also examine the RS spectra with respect to the different meningioma subtypes. With our classifier, we are not able to distinguish them. By using more sophisticated machine learning algorithms, it may be possible in the future to discriminate between the different meningioma subtypes and also use the tool for tumor diagnostics. However, the fact that the meningioma subtypes share many spectral features when compared to dura mater is advantageous for the purpose of the tool as outlined here, as it is not critical to know the type of meningioma before analysis.

While metallic aluminum has a negligible Raman spectrum, its oxidized form shows a spectrum below 900 cm^−1^^34,35^. We excluded this interval of small wavenumbers in our analyses as we noticed the influence especially in smaller samples. Other substrates with lower spectral interference exist, i.e., calcium fluoride (CaF_2_) with only one Raman peak at 322 cm^−1^^36^. CaF_2_ is particularly suited for microscopic applications as it is translucent. In this study, we chose aluminum for its low intrinsic signal and good availability in the surgery room.

If applied on the surgical-site-simulating verification sample, our trained classifier was able to distinguish tumor from healthy areas of the sample that were different in histology but indistinguishable macroscopically. Even small clusters of tumor cells inside the dura mater are identified by the classifier (Fig. 5). A classification of infiltration zone as dura based on a single spot measurement would be problematic. From the surgeon’s view, the accuracy of the classifier depends on the desired information: For the single spot measurement the classifier can be assumed to be true regarding the nearly 100% sensitivity but for a spacious evaluation of the infiltration zone, the accuracy will decrease because of the inherent nature of the infiltration zone. The infiltration zone contains tumor cell clusters in widespread distances whereas the rest consists of healthy dura mater. The challenge will be to detect exactly these tumorous areas, which leads automatically to a higher number of needed measurements per area unit. With single measurements, taking no longer than a few seconds, the surgeon will be able to identify the pure tumor and dura mater regions. By measuring point-by-point the surgeon receives direct feedback on the dignity of the tissue and thus he discovers whether he needs to resect further at this site. For proper detection of the extent of the infiltration zone, the number of measurements needs to be increased in a systematic way. This can be achieved by systematic scanning of the surgery site and potentially assisted by a robot-navigated probe holder that is integrated into the neuronavigation system. Complete mapping of the infiltration zone cannot be achieved because the infiltration zone is always of a three-dimensional extent in space but the scan is performed on the surface only.

## Conclusion

Since tumor residues are often the reason for recurrence/progression of meningiomas, an appropriate, fast and reliable intraoperative tool to discriminate between neoplastic and healthy meningeal tissue is crucial. In this study, we present a new intraoperative RS application without the need of preanalytical tissue processing. The method complements the neuropathological analysis and extends it into the surgery room. It is less time-consuming, can be applied by non-neuropathologically-specialized staff and is therefore less expensive. The application has the potential to be included into the daily neurosurgical routine in the near future to aid residue-free resection of tumors, e.g. meningiomas. To ensure a smooth transition from the ’classical’ neuropathological method to RS-based tumor-identification and removal, the preliminary diagnosis gained with the spectroscopic classifier should always be verified by a neuropathologist and, of course, a contrasted CT or MRI shall be done afterwards to verify a Simpson grade I resection. Our future goal is to extend RS-based tumor detection to further neuronal tissue entities i.e. to glioblastomas. Macroscopic RS could also lead to biochemical insights in tumor biology, as a direct and non-destructive assessment of Raman active metabolites and structural elements can be recorded. This may help to identify the most suitable personalized therapy of meningiomas and to predict the effectiveness of upcoming target therapies in the future^37^.

In summary, we show that Raman spectroscopy can differentiate macroscopically between meningioma and normal dura mater. This is verified by means of neuropathological histological assessment. A serial of systematic scanning of the tissue surface can reliably identify the tumor as well as infiltrated tissue if enough scans are done per area unit. The classifier visualization design with an overlaid transparent heat map on the macroscopic image (Fig. 5) might be applicable for the fade-in into a surgical microscope in the future. In vivo, a robotic device might be used to aid in systematic scanning of the infiltration zone. For the direct application in the operating field, a hand-held RS probe (integrated in a neuronavigation system and/or in the surgical microscope) might be available in the future^14^.

## Supplementary Information

Supplementary Information.

## Data Availability

The ethical approval does not permit public sharing of data. Interested parties are invited to contact the corresponding author for individual options.
