# Supplementary material for: Intraoperative discrimination of native meningioma and dura mater by Raman spectroscopy
Source: Sci Rep. 2021 Dec 8;11:23583. doi: 10.1038/s41598-021-02977-7 (PMC8654829; doi:10.1038/s41598-021-02977-7)
Supplement: Supplementary file 1 — Supplementary Information. [file 41598_2021_2977_MOESM1_ESM.docx]

Supplements


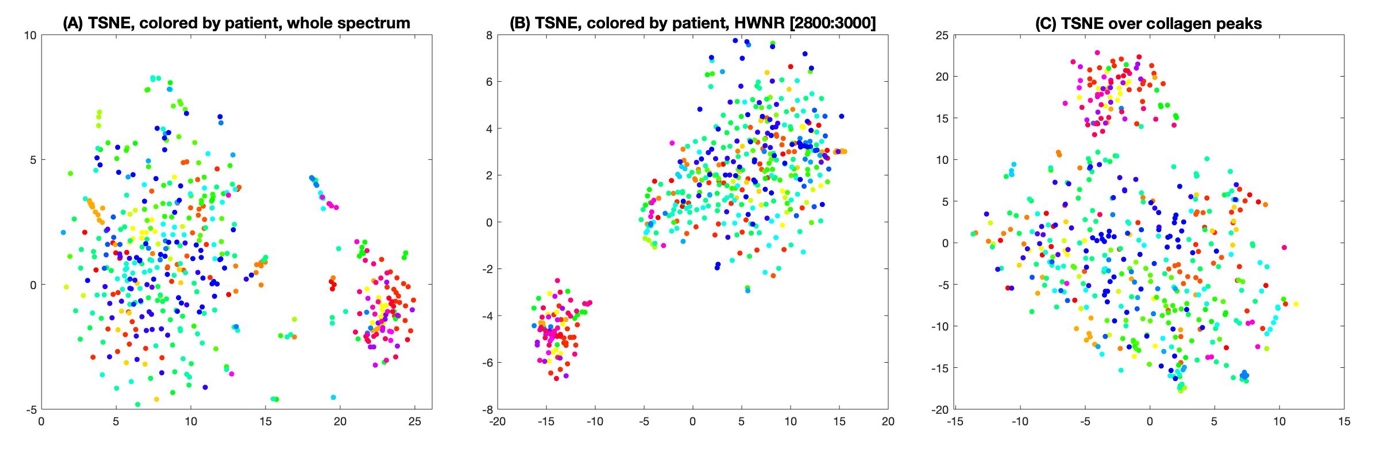


**Supplementary Figure 1.** tSNE-Cluster of meningioma subtypes and dura mater *patient-based* to rule out clustering by patient due to some potential hidden similarities for each patient **(A)**, tSNE-Cluster of pathologically secured diagnoses of entire Raman spectrum of the chosen subclasses. **(B)** tSNE Cluster of pathologically approved diagnoses over high-wavenumber region (HWNR), interval 2800 *cm^−1^* to 3000 *cm^−1^*. **(C)** tSNE Clusters based on collagen peaks


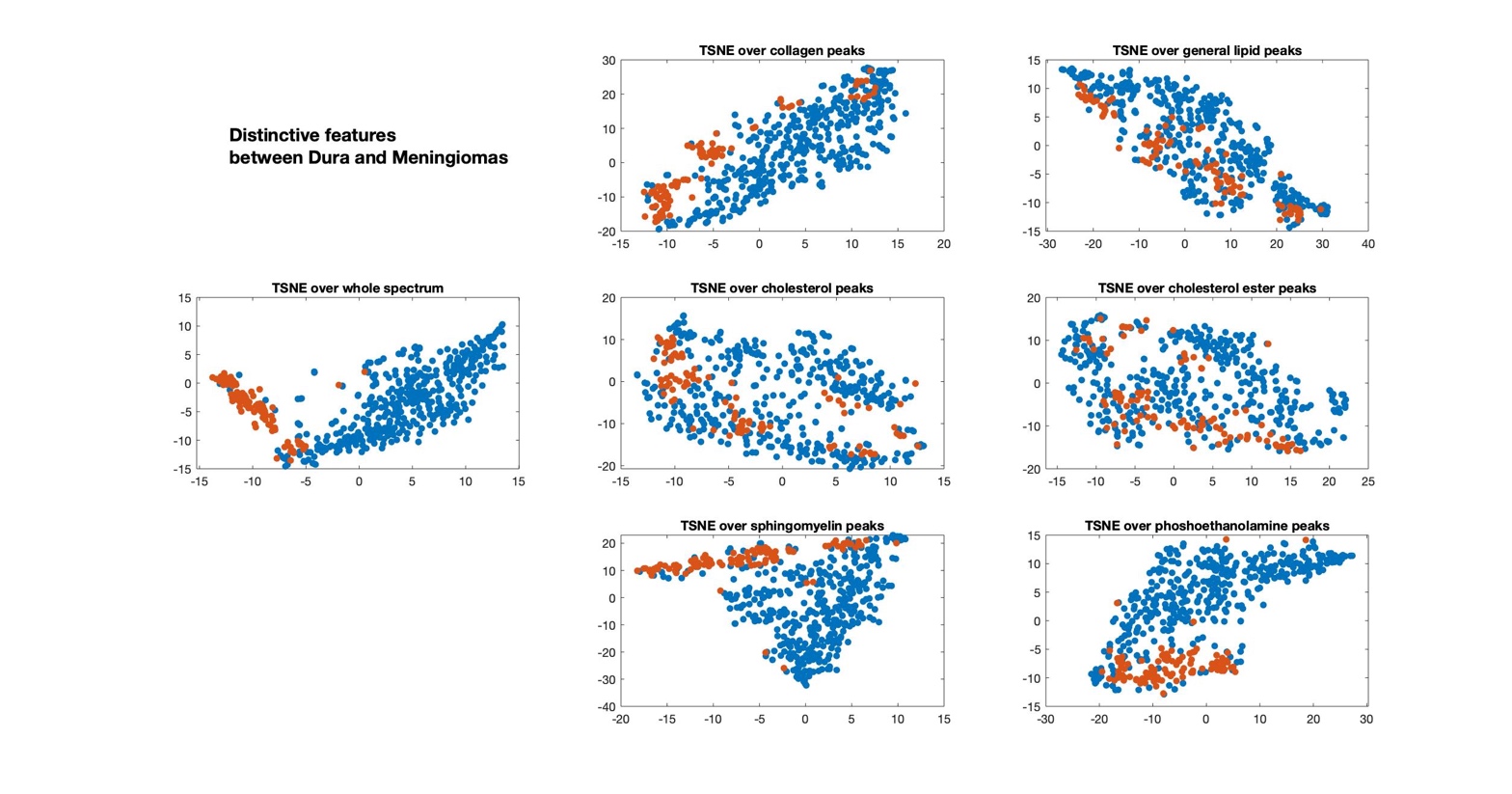


**Supplementary Figure 2.** tSNE-Cluster of meningioma subtypes (blue dots) and dura mater (red dots) clustered over specific peaks:

-**collagen peaks:** 815cm^-1^ ,855 cm^-1^, 876 cm^-1^, 938 cm^-1^, 1003 cm^-1^, 1033 cm^-1^, 1250 cm^-1^, 1267 cm^-1^, 1319 cm^-1^, 1450 cm^-1^, 1663 cm^-1^

-**general lipid peaks:** 1260 cm^-1^,1300 cm^-1^, 1440 cm^-1^, 1656 cm^-1^, 1734 cm^-1^

-**cholesterol peaks:** 418 cm^-1^, 421 cm^-1^, 457 cm^-1^, 549 cm^-1^, 608 cm^-1^, 700 cm^-1^, 702 cm^-1^, 703 cm^-1^, 746 cm^-1^, 759 cm^-1^, 853 cm^-1^, 881 cm^-1^, 957 cm^-1^, 962 cm^-1^, 1132 cm^-1^, 1179 cm^-1^, 1300 cm^-1^, 1440 cm^-1^, 1441 cm^-1^, 1444 cm^-1^, 1659 cm^-1^, 1661 cm^-1^, 1670 cm^-1^, 1674 cm^-1^, 2970 cm^-1^

-**cholesterol ester peaks:** 428 cm^-1^, 538 cm^-1^, 614 cm^-1^, 702 cm^-1^, 1065 cm^-1^, 1131 cm^-1^, 1296 cm^-1^, 1441 cm^-1^, 1669 cm^-1^, 1670 cm^-1^, 1739 cm^-1^, 2970 cm^-1^

-**sphingomyelin peaks:** 1437 cm^-1^, 1654 cm^-1^, 1670 cm^-1^, 2847 cm^-1^, 2880 cm^-1^, 2959 cm^-1^

-**phosphoethanolamine peaks:** 760 cm^-1^, 1442 cm^-1^, 1657 cm^-1^, 1737 cm^-1^, 2847 cm^-1^, 2882 cm^-1^, 2920 cm^-1^, 2959 cm^-1^, 3007 cm^-1^


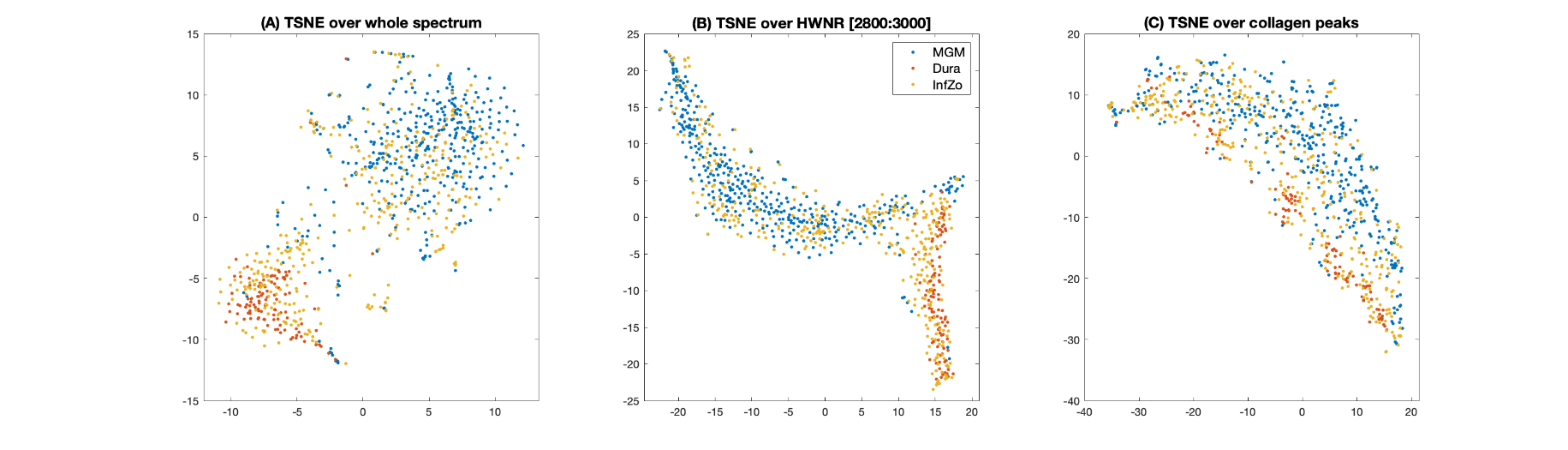


**Supplementary Figure 3.** tSNE-Cluster of meningioma subtypes (blue dots) and dura mater (red dots) and infiltration zone (yellow dots). Infiltration zone samples that are histopathologically shown to be heterogenous cluster with either meningioma or dura mater spectra.


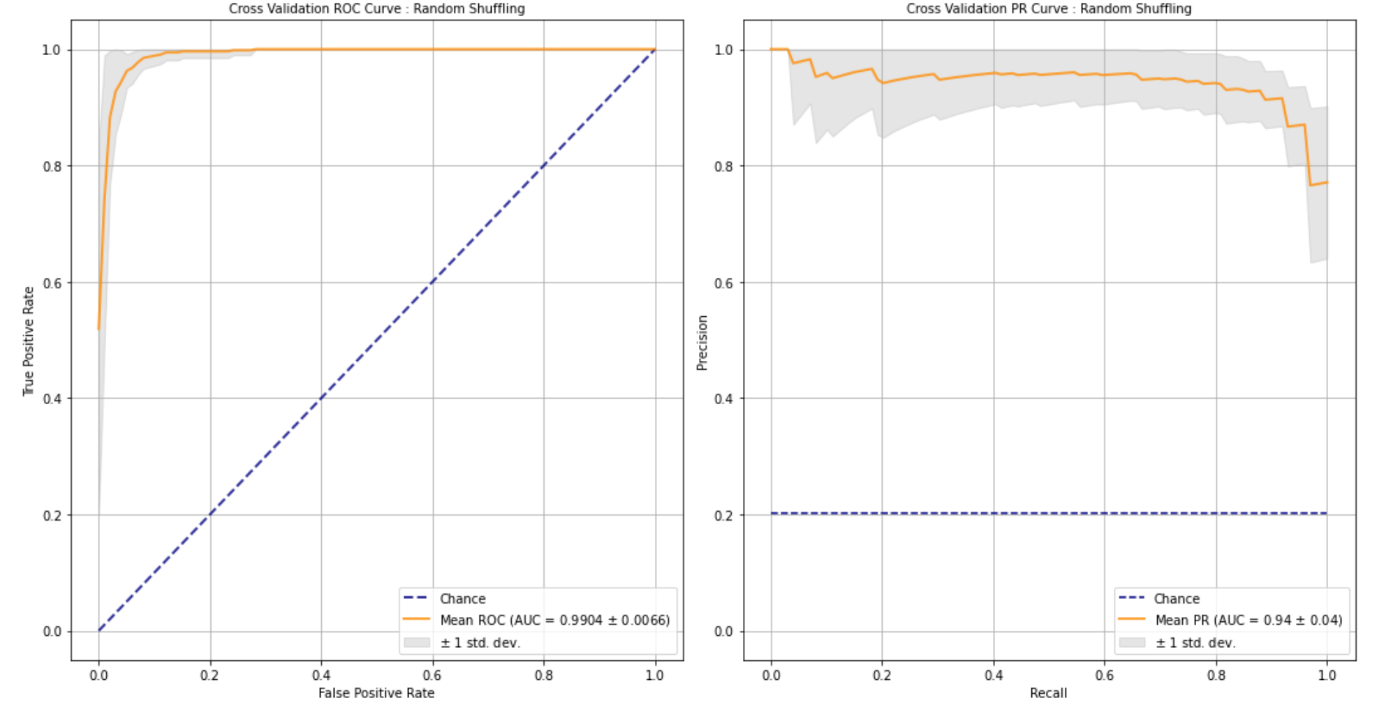


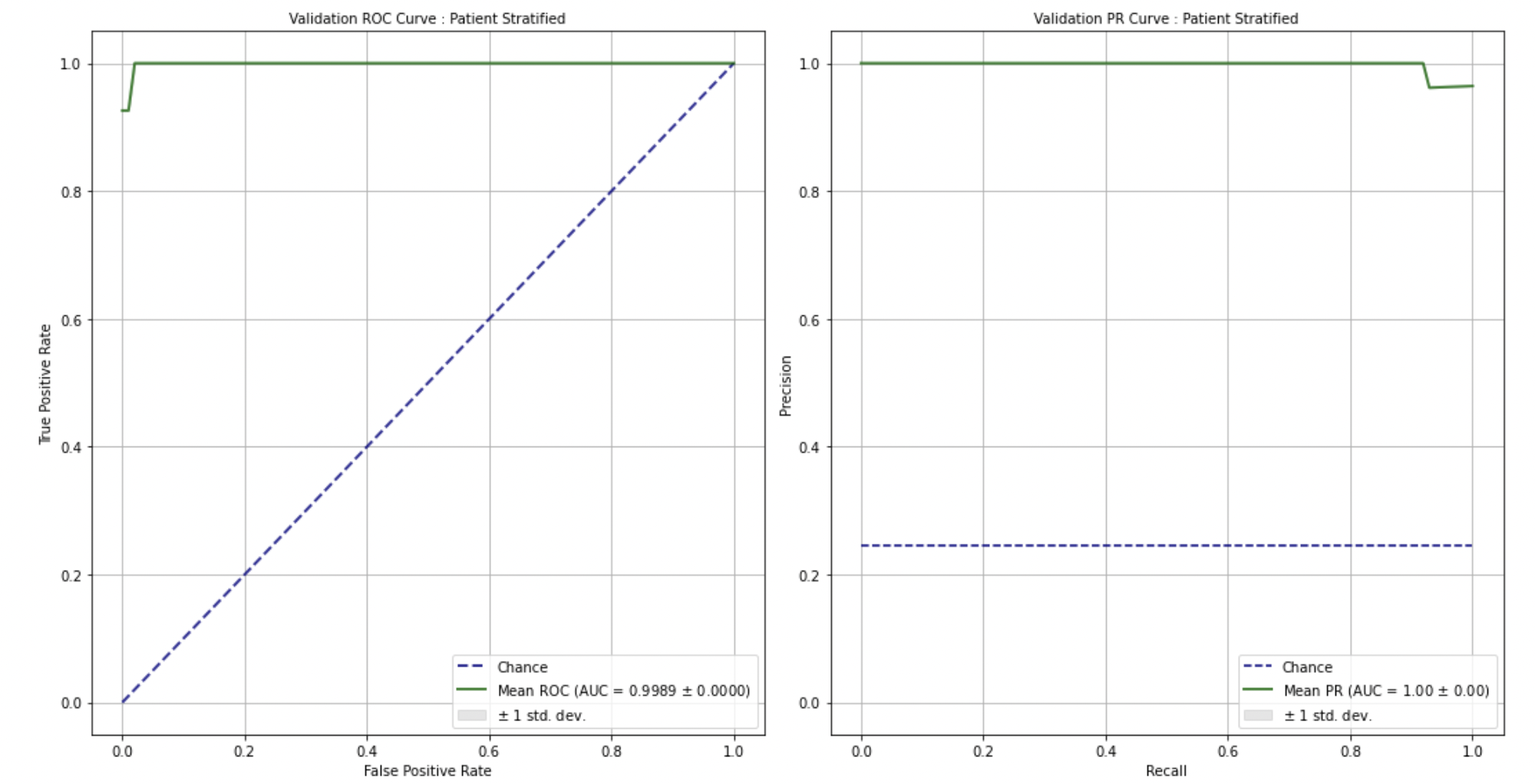


**Supplementary Figure 4.** ROC and PR for the cross validation dataset (orange) and external test set (green). Chance level is indicated with the dotted line. At the optimal threshold (best g-mean score) of the training set, the performance metrics are as follows. Training set: 96.06 $\pm$ 0.03% sensitivity, 95.44 $\pm$ 0.02% specificity, 84.85 $\pm$ 0.06% precision, 95.57 $\pm$ 0.02% accuracy, 95.75 $\pm$ 0.02% balanced accuracy; 0.98 $\pm$ 0.01 AUROC, 0.94 $\pm$ 0.4 AUPR ; External test set: 100% sensitivity, 93.97% specificity, 84.37% precision, 95.45% accuracy, 96.98% balanced accuracy, 0.99 AUROC, 0.99 AUPR.

|  | **Peaks (cm^-1^)** | **Assignments** | **Literature** |
| --- | --- | --- | --- |
| **Dura mater/collagen** | **815** | **(hydroxy-) proline** | **Cheng et al. 2005**^1^ |
| **Dura mater/collagen** | **852** | **Proline, hydroxyproline, tyrosine** | **Cheng et al. 2005**^1^ |
| **Dura mater/collagen** | **855** | **(hydroxy-) proline** | **Huang et al. 2003**^2^ |
| **Dura mater/collagen** | **876** | **(hydroxy-) proline** | **Huang et al. 2003**^2^  **Frank et al. 1995**^3^ |
| **Dura mater/collagen** | **938** | **(hydroxy-) proline** | **Cheng et al. 2005**^1^ |
| **Dura mater/collagen** | **1003** | **phenylalanine** | **Chan et al. 2006**^4^ |
| **Dura mater/collagen** | **1033** | **phenylalanine** | **Chan et al. 2006**^4^ |
| **Dura mater/collagen** | **1245** | **Amide III** | **Shetty et al. 2006**^5^ |
| **Dura mater/collagen** | **1250** | **amide III** | **Cheng et al. 2005**^6^ |
| **Dura mater/collagen** | **1267** | **amide III** | **Malini et al. 2006**^7^ |
| **Dura mater/collagen** | **1271** | **Amide III band in proteins** | **Sigurdsson et al. 2004**^8^ |
| **Dura mater/collagen** | **1319** | **CH2CH3 deformation** | **Frank et al. 1995**^3^ |
| **Dura mater/collagen** | **1447** | **CH2 bending mode of proteins & lipids** | **Faolain et al. 2005**^9^ |
| **Dura mater/collagen** | **1450** | **CH2CH3 deformation** | **Shafer-Peltier et al. 2002**^10^ |
| **Dura mater/collagen** | **1637** | **Amid I** | **Faolain et al. 2005**^9^ |
| **Dura mater/collagen** | **1661** | **Amide I band** | **Naumann et al. 1998**^11^ |
| **Dura mater/collagen** | **1663** | **proteins, collagen type I** | **Binoy et al. 2004**^12^ |
| **Dura mater/collagen** | **2940** | **C-H vibrations in lipids & proteins** | **Sigurdsson et al. 2004**^8^ |

**Supplementary Table1:** Main collagen/ dura mater peaks highlighted in the manuscript with references.

**
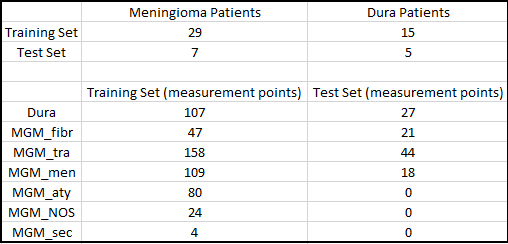
**

**Supplementary Table 2: Modeling data distribution**
